# Cell-cell contact-driven EphB1 cis- and trans- signalings regulate cancer stem cells enrichment after chemotherapy

**DOI:** 10.1038/s41419-022-05385-5

**Published:** 2022-11-19

**Authors:** Lujuan Wang, Qiu Peng, Yaohuan Xie, Na Yin, Jiaqi Xu, Anqi Chen, Junqi Yi, Wenhua Shi, Jingqun Tang, Juanjuan Xiang

**Affiliations:** 1 Hunan Cancer Hospital, the Affiliated Cancer Hospital of Xiangya School of Medicine, Central South University, Changsha, Hunan PR China; 2 Cancer Research Institute, School of Basic Medical Science, Central South University, Changsha, Hunan China; 3 NHC Key Laboratory of Carcinogenesis and the Key Laboratory of Carcinogenesis and Cancer Invasion of the Chinese Ministry of Education, Xiangya Hospital, Central South University, Changsha, Hunan China; 4 Hunan Key Laboratory of Tumor Models and Individualized Medicine, Department of Orthopaedics, The Second Xiangya Hospital, Central South University, Changsha, China; 5 Department of thoracic surgery, the Second Xiangya Hospital, Central South University, Changsha, Hunan 410013 China; 6 Hunan Key laboratory of Early Diagnosis and Precise Treatment of Lung Cancer, the Second Xiangya Hospital, Central South University, Changsha, 410013 Hunan China

**Keywords:** Non-small-cell lung cancer, Epithelial-mesenchymal transition

## Abstract

Reactivation of chemotherapy-induced dormant cancer cells is the main cause of relapse and metastasis. The molecular mechanisms underlying remain to be elucidated. In this study, we introduced a cellular model that mimics the process of cisplatin responsiveness in NSCLC patients. We found that during the process of dormancy and reactivation induced by cisplatin, NSCLC cells underwent sequential EMT-MET with enrichment of cancer stem cells. The ATAC-seq combined with motif analysis revealed that OCT4-SOX2-TCF-NANOG motifs were associated with the enrichment of cancer stem cells induced by chemotherapy. Gene expression profiling suggested a dynamic regulatory mechanism during the process of enrichment of cancer stem cells, where Nanog showed upregulation in the dormant state and SOX2 showed upregulation in the reactivated state. Further, we showed that EphB1 and p-EphB1 showed dynamic expression in the process of cancer cell dormancy and reactivation, where the expression profiles of EphB1 and p-EphB1 showed negatively correlated. In the dormant EMT cells which showed disrupted cell-cell contacts, ligand-independent EphB1 promoted entry of lung cancer cells into dormancy through activating p-p38 and downregulating E-cadherin. On the contrary, in the state of MET, in which cell-cell adhesion was recovered, interactions of EphB1 and ligand EphrinB2 in trans promoted the stemness of cancer cells through upregulating Nanog and Sox2. In conclusion, lung cancer stem cells were enriched during the process of cellular response to chemotherapy. EphB1 cis- and trans- signalings function in the dormant and reactivated state of lung cancer cells respectively. It may provide a therapeutic strategy that target the evolution process of cancer cells induced by chemotherapy.

## Introduction

Lung cancer is the most common type of cancers around the world. Cisplatin-based chemotherapy is the first-line therapeutic management for non-small cell lung cancer (NSCLC). Despite the therapeutic benefits, relapse following chemotherapy induced-remission remains a problem. Selective pressure from chemotherapy directs the evolution of cancer cells. A significant enrichment for dormant cells that are refractory to treatment has been found in cancer biopsies after exposure to chemotherapy [1, 2]. After a long-term dormancy, cancer cells regrow and cause metastatic relapse. The existence of dormant cancer cells increases the risk of relapse. Cellular dormancy is characterized by non-dividing cells with low levels of ki67 and cell cycle arrest in G0/G1 [3]. Dormant cells also show the traits of epithelial- mesenchymal transition (EMT) [4]. EMT is an essential phenotypic process that epithelial cells acquire fibroblast-like properties and show reduced cell-cell adhesion and higher drug resistance [5]. The inactivation of two of the most-studied pathways RAS-MEK-ERK/MAPK and PI3K-AKT plays a critical role in governing cancer cell dormancy [4]. Cancer stem cells (CSCs) share overlapping characteristics and signalings with dormant cancer cells including therapy resistance [6]. Dormancy, therapy resistance, EMT and plasticity of CSCs are interconnected processes [6]. A better understanding the mechanisms of chemotherapy-induced cellular dormancy and relapse helps to develop therapeutic strategies to overcome the challenges of chemotherapy-driven cancer progression.

Ephrin receptors (Ephs) form the largest group of RTKs. Eph receptors family is tyrosine kinases and their binding with ligands transduce signaling in both receptor- and ligand-expressing cells [7]. Eph genes are frequently mutated in lung adenocarcinoma and mutations of EphB1 are associated with copy number gain [8]. Eph signaling is important repulsive signals that counteract the adhesion between cells during development, cell adhesion, migration, repair after nervous system injury, and maintenance of gap junctions [9]. Eph receptors have been found to play an important role in EMT [10, 11]. EphB3 interacts with E-cadherin and induces shedding of E-cadherin by ADAM10 [12]. EphA2 promotes EMT through β-catenin in gastric cancer cells [13]. On the other hand, ligand-activated EphA2 enhances cell-cell adhesion and promotes MET, the reverse process of EMT in corneal epithelial cells [14]. EphB1 has both tumor-suppressing and tumor-promoting roles in some kind of tumors [15, 16]. EphB1 has been found to be upregulated in lung cancer biopsies compared to non-cancer controls [17]. In contrast to other RTKs that bind soluble ligands, Eph receptors bind membrane bound ligands and drive bidirectional signaling [7]. The activation of Eph receptors upon membrane bound ephrins resembles those used by other RTKs whose receptors undergo heterodimerization for activation [18]. However, a more complex situation is encountered in cells co-expressing both receptors and ephrins [19]. The Eph receptors can oligomerize to form clusters via direct Eph/Eph interactions without Ephrin ligands [18]. The ligand-binding domain (LBD) and cysteine-rich domain (CRD) domain are dimerizing determinant allowing Eph-Eph interaction [18, 20]. The roles of fibronectin III domain in the clustering interfaces have not been confirmed [21]. It is commonly assumed that trans interactions with Ephrin ligands which happen between two adjacent cells are Eph forward signal activating, while cis interactions which happen within the same cells makes Eph receptors less responsive to Ephrin ligands and abolish forward tyrosine phosphorylation signaling [22–24].

In many contexts, trans signal between opposing cells is critical. However, the extent to which cis signaling mode are utilized may represent important regulatory roles [25]. Our previous study has demonstrated the pro-invasive roles of ligand independent EphB1 signaling [17]. In this study, we introduced a cellular model that mimics the process of cisplatin responsiveness in NSCLC patients. We found that cisplatin induced the enrichment of cancer stem cells through the process of dormancy and reactivation. We found that EphB1 ligand-independent and -dependent signaling can be attributed to E-cadherin-induced cell-cell adhesion. Ligand-independent EphB1 promoted entry of lung cancer cells into dormancy through activating p-p38 and downregulating E-cadherin. On the contrary, interactions of EphB1 and ligand ephrinB2 in trans promoted the stemness of cancer cells through upregulating Sox2 during the shift from cellular dormancy to reactivation. Our results indicate that EphB1 ligand dependent and independent signaling play pivotal roles in regulating tumor dormancy and reactivation.

## Results

### Cisplatin induces cellular dormancy and reactivation and EMT-MET transition

**Fig. 1:**
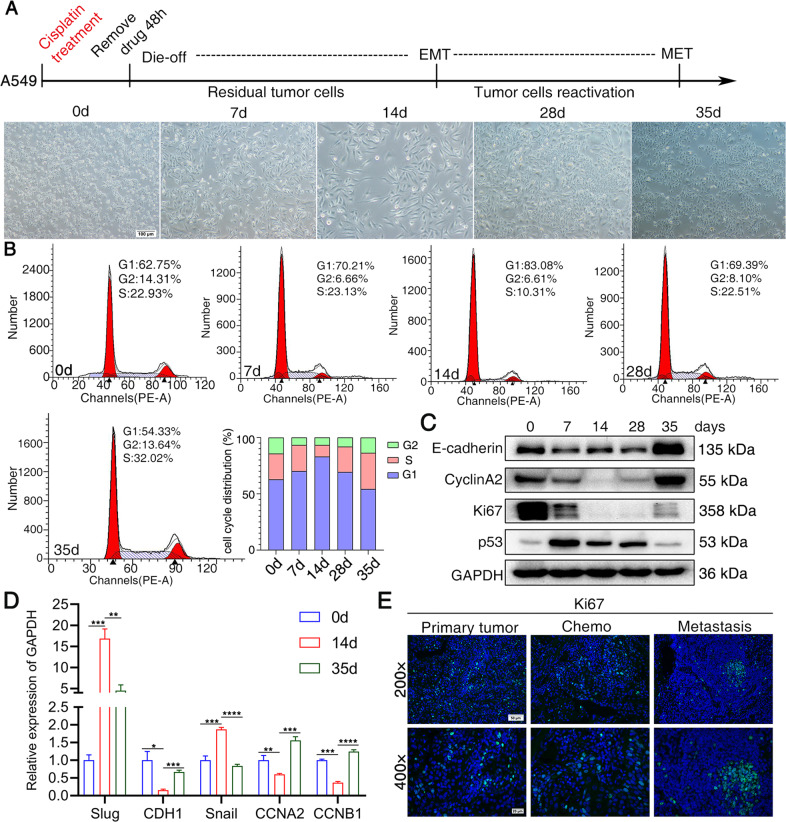
Cisplatin induces cellular dormancy and reactivation and EMT-MET transition. NSCLC cells were subjected to short-term exposure to cisplatin. Lung cancer cells were treated with cisplatin for 48 h. Cisplatin was then withdrawn. **A** The treatment protocol and the microscope images of A549 cells treated with cisplatin at different time points; **B** Cell cycle analysis of dormant and reactivation cancer cells induced by cisplatin; **C** Western blot analysis of proteins obtained from cisplatin-treated A549 cells at different time points; **D** RT-qPCR analysis of mRNA obtained from cisplatin-treated A549 cells at different time points; Data are presented as the mean ± S.D. from at least three separate experiments. Student’s *t* test, *P value < 0.05, **P value < 0.01, ***P value < 0.001, ****P value < 0.0001; **E** Assessment of Ki67 in lung cancer patients. Tumor biopsies were fixed and stained for fluorescence labeled Ki67. Scale bar = 50 μm. Primary tumor: biopsies from lung primary tumors; Chemo: biopsies from lung tumors after neoadjuvant chemotherapy; Metastasis: biopsies from metastatic brain of lung cancer patients;.

Conventional chemotherapy initially kills most cancer cells; however, the residual cancer cells eventually survive and cause metastatic relapse. To evaluate the phenotype of chemotherapy-treated cells, we introduced here an in vitro model of tumor dormancy and recurrence induced by short-term treatment of cisplatin according to previously published protocols [26–28]. Lung cancer cells were subjected to short-term exposure to cisplatin. The treatment protocol was summarized in Fig. 1A. Forty-eight hours after treatment, most of the cells were killed, whereas the surviving cells acquired a fibroblastoid-like cell shape. The cisplatin was then withdrawn. About 28 days after the removal of cisplatin, the cells returned to a polygonal epithelial-like state (Fig. 1A, Supplementary Fig. 1a, b). Most cells that survive the chemotherapy entered into G0-G1 phase (83.08% of cell cycle in 14 days after cisplatin withdrawal), with low expression of proliferative marker ki67 from 7 to 14 days after cisplatin treatment (Fig. 1B, C, Supplementary Fig. 1c), which indicated that the cells were in dormant state. After several days of quiescence, cells regrew and reentered into S phase, showing high expression of ki67, suggesting that cells recovered from dormant state (Fig. 1B, C) . The dynamic expression of E-cadherin, CyclinA2, p53 and ki67 throughout the cellular process were shown by western blot (Fig. 1C). Enhanced mRNA expressions for mesenchymal markers slug and snail and decreased expression of epithelial marker E-cadherin were observed in dormant cancer cells (Fig. 1D). The cell cycle markers CCNA2 and CCNB1 showed altered expressions in correspondence to the dormant and reactivated states (Fig. 1C, D). We collected cancer biopsies from patients who underwent surgery after neoadjuvant chemotherapy and brain metastatic samples of lung cancer patients. The dormant and reactivated status were confirmed clinically by immunofluorescent staining of ki67 (Fig. 1E, Supplementary Fig. 1d).

### Cisplatin-induced dormant and reactivated lung cancer cells show distinct gene expression profile

**Fig. 2:**
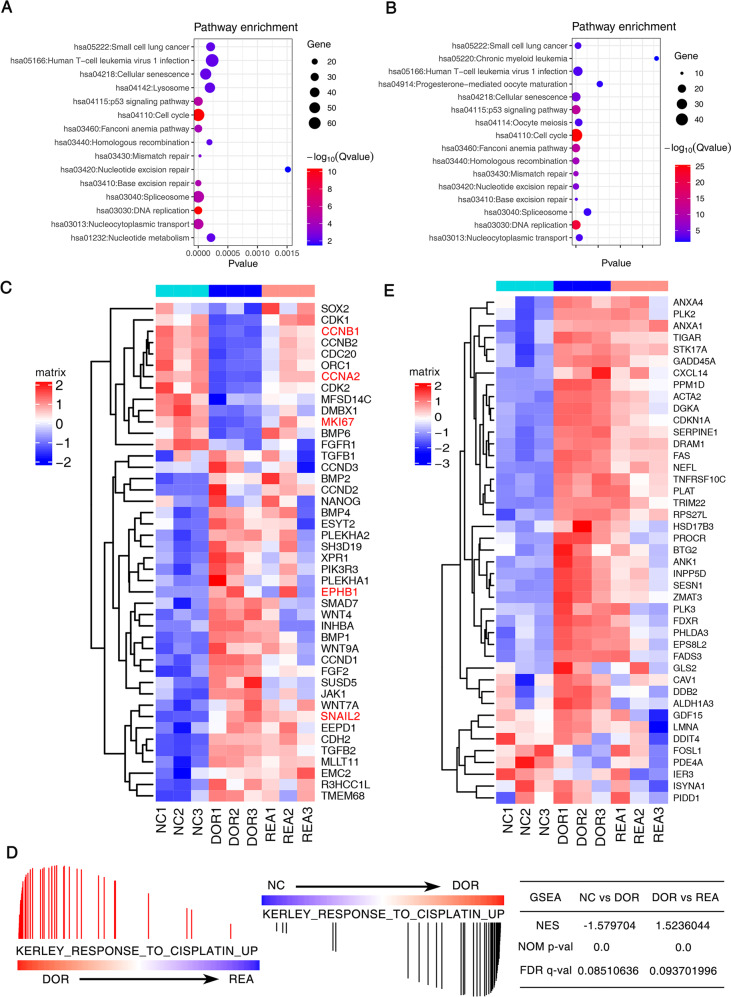
Cisplatin-induced dormant and reactivated lung cancer cells show distinct gene expression profiles. The differential gene expression profiles were evaluated by RNA-seq. The DEGs were subjected to KEGG pathway enrichment analysis between dormant cancer cells and untreated cancer cells (**A**); between reactivated cancer cells and dormant cancer cells (**B**); **C** The heatmap of the expression of significant genes in cell cycle, EMT et al. between 3 cell phases induced by cisplatin; **D** GSEA analysis of differentially expressed genes. Gene set enrichment analysis using “GSEA, KERLEY_RESPONSE_TO_CISPLATIN_UP” was performed. *P* value < 0.001. The NES and FDR were shown. **E** The heatmap of the expression of significant genes in cisplatin-resistance between 3 cell phases induced by cisplatin.

To investigate the differential gene expression profiling between the untreated lung cancer cell, cells that survived the cisplatin and cells regrew after long period of quiescence, we performed RNA-sequencing on cells with these 3 states (NC, DOR and REA). The sequencing data was filtered and the clean reads were mapped to the reference genome (Homo_sapiens, GCF_000001405.38_GRCh38.p12). The genome mapping ratio was from 87.05 to 92.03% (Supplementary Fig. 2a). There were 3834 differentially expression genes (DEGs) between dormant cancer cells and NC cancer cells and 814 DEGs between reactivated cancer cells and dormant cancer cells, indicating that cells underwent less changes in activated process compared to dormant process (*P* < 0.05, Supplementary fig. 2b, c). Pearson’s correlation coefficient of gene expression among 3 groups was plotted as a clustered heatmap, where the colors represented the correlation coefficients. It automatically led to a clustering and revealed some similarity between dormant and reactivated cancer cells, indicating that reactivated cancer cells shared some characteristics of dormant cancer cells (Supplementary fig. 2d). The DEGs with most fold change value including CCNB2, CDC6, IGFBP7, ACTA2 et al. were shown in Supplementary fig. 2e. All the 3834 DEGs were subjected to KEGG pathway enrichment analysis. A *P* value was calculated by Phyper for a hypergeometric distribution. A Q value was determined by adjusting *P* value for the False Discovery Rate (FDR). As shown in Fig. 2A and B, pathway enrichment with significant difference (*Q* value < 0.05) indicated that DEGs between 3 states of cancer cells included the genes related to DNA replication, cell cycle (Fig. 2A, B). The heatmap revealed that the significant differences between these 3 phase cells involved in cell cycle related genes CDK1, CDK2, CCNA2, EMT related genes TGF-β, SNAIL2, CDH2, EphB1 et al. (Fig. 2C). Pathway analyses using the Gene Set Enrichment Analysis (GSEA) program revealed that gene sets such as cell proliferation, cell cycle cell adhesion and p53 pathway were significantly dynamically altered in dormant and reactivated lung cancer cells (Supplementary Fig. 2f). As expected, we observed cisplatin resistant genes ANAX1, PLK2, ANXA1 et al. (GSEA, KERLEY_RESPONSE_TO_CISPLATIN_UP) to be dynamically expressed during the process of dormancy and reactivation (Fig. 2D). The heatmap revealed the significant differences in cisplatin-responsive genes between these 3 phase cells (Fig. 2E).

### The process of dormancy and reactivation induced by chemotherapy enriches cancer stem cells

**Fig. 3:**
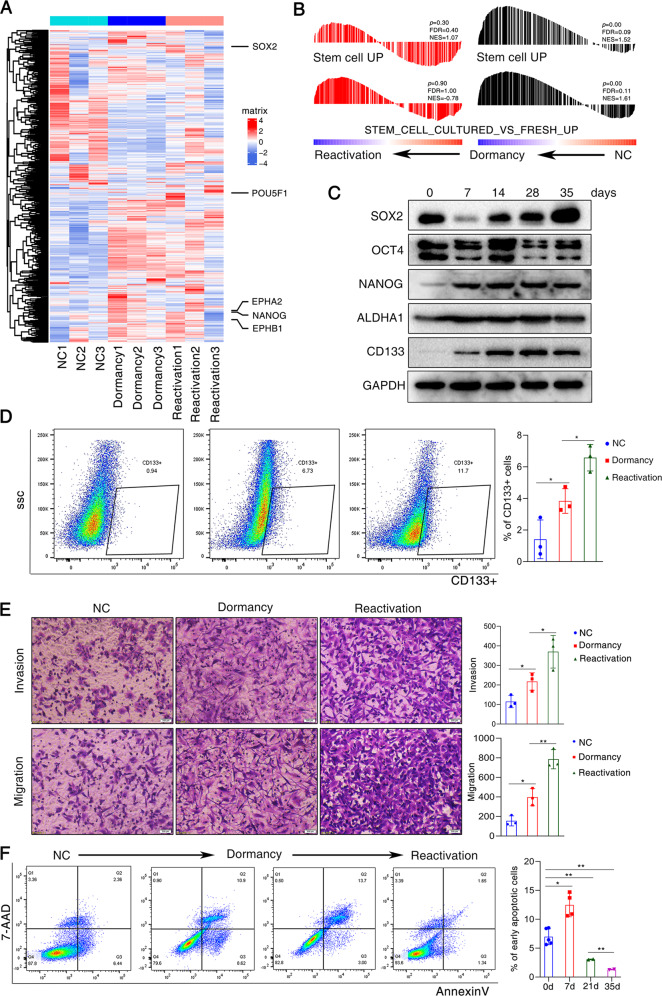
The process of dormancy and reactivation induced by chemotherapy enriches cancer stem cells. **A** The heatmap of the expression of stem-cell-related genes with significant difference between 3 cell phases induced by cisplatin. Pou5F1 (Oct4), Sox2 and Nanog were shown in figure; **B** GSEA analysis of differentially expressed genes. Gene set enrichment analysis using “stem cell” was performed. *p* < 0.001; The NES and FDR were shown. **C** Western blot analysis of proteins obtained from cisplatin-treated A549 cells at different time points; **D** Flow cytometry analysis for stem cell surface marker CD133 in the 3 stages of cells. The histogram graph was shown on the right. Data are presented as the mean ± S.D. from at least three separate experiments. **E** Transwell migration and invasion assays. Representative microscopic images of cells in the 3 stages that migrated through or invaded through the transwell were shown. The histogram graph was shown on the right. Data are presented as the mean ± S.D. from at least three separate experiments. **F** Flow cytometry analysis of apoptotic cells after treatment of cisplatin. Representative flow cytometry plots using Annexin V-FITC/PI staining for apoptosis. The percentage of apoptotic cells were shown on the right. Data are presented as the mean ± S.D. from at least three separate experiments. Student’s *t* test, *P value < 0.05, **P value < 0.01, ***P value < 0.001.

To specifically investigate if these 3 phase cells differ in regard to the expression of stemness related genes, we analyzed the stem-cell-related genes and their differentiated counterparts (Gene sets: GSEA BEIER-STEM CELL, GSEA BOQUEST-STEM CELL). We found that the expressions of stemness related genes showed upregulated during the process of cisplatin-induced dormancy and reactivation (Fig. 3A, B). Enhanced expressions for cancer stem cell markers Sox2, POU5F1(Oct4), Nanog, ALDHA1 and CD133 were observed in dormant and reactivated cancer cells after treatment of cisplatin (Fig. 3C, D). Of note, the expression patterns of Sox2 were different from those of Nanog and CD133 (Fig. 3C). It suggested that transcription factors such as Sox2 and Nanog play roles in different phases of cancer stem cell enrichment. The dormant and reactivated cancer cells showed enhanced migration, invasion and drug resistance (Fig. 3E, F), suggesting the cancer stem cell properties in these 2 phase cells. Of interest, although the cells underwent EMT and MET and the expression of E-cadherin recovered, the molecular expression profiles and behaviors do not recover to the intact status, while the reactivated cancer cells showed more cancer stem cell-like characteristics.

### OCT4-SOX2-TCF-NANOG were enriched in dormant and reactivated cancer cells

**Fig. 4:**
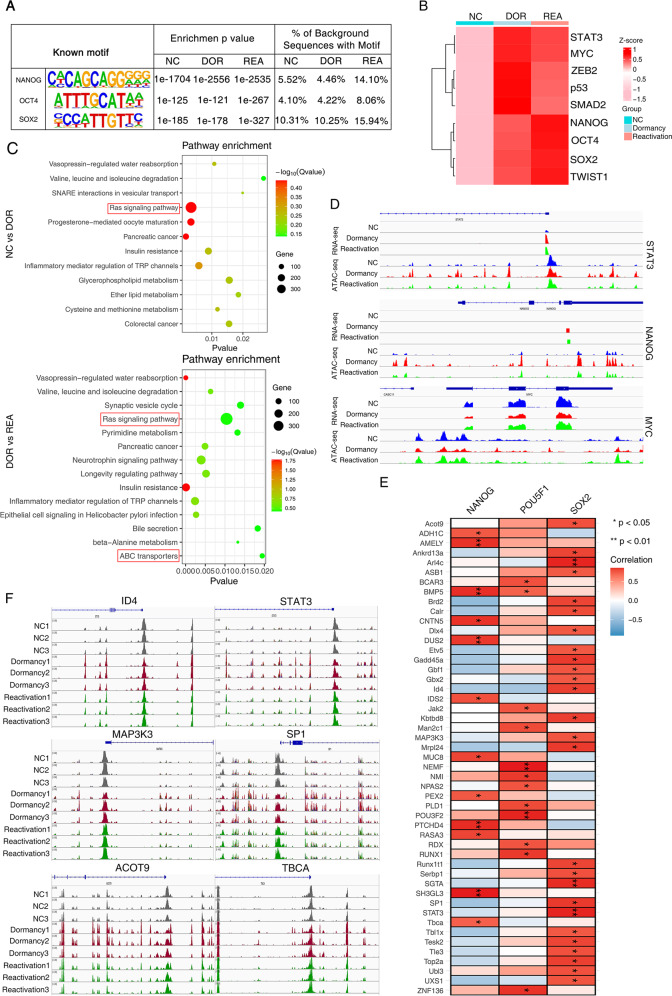
OCT4-SOX2-TCF-NANOG is enriched in dormant and reactivated cancer cells. The open chromatin in cells were evaluated by ATAC-seq. **A** HOMER motif analysis for transcription factor binding sites in regions of open chromatin. The frequency of transcription factor binding sites of Oct4, Nanog and Sox2 within the open chromatin was expressed as a percentage. The motif enrichment *P*-value was indicated. **B** The accessibility of representative motifs (mean motif enrichment *P* value) among ATAC-seq of the 3 cell phases induced by cisplatin. **C** The differential peaks were subjected to KEGG pathway enrichment analysis between dormant cancer cells and untreated cancer cells (upper); between reactivated cancer cells and dormant cancer cells (below); **D** The integrative genomics viewer (IGV) visualization of RNA-seq and ATAC-seq near STAT3, Nanog, Myc gene loci in 3 cell phases. **E** Spearman’s correlation analysis. The correlation between the promoter accessibility of stem cell-related genes and expressions of Nanog, Oct4 and Sox2 was evaluated, *P < 0.05. **F** The genomic profiles of specific differentially accessible regions of representative target genes of Nanog, Oct4 and Sox2 were visualized by integrative genomics viewer (IGV).

To identify regulatory factors that mediate cancer cell state reprogramming induced by chemotherapy, we interactively analyzed the profiles from an assay for transposase-accessible chromatin sequencing (ATAC-seq) and RNA sequencing (RNA-seq) in dormant and reactivated A549 cells. ATAC-seq profiles revealed that dormant and reactivated lung cancer cells possessed more open chromatin regions than untreated control cells. On average, we identified 108,407 accessible peaks in dormant cells and 107,898 accessible peaks in reactivated cells, which possessed more open chromatin regions than untreated control cells (peak number = 72,309). Peaks were classified based on the location (UCSC annotation data) and showed the genome regions including intergenic, introns, downstream, upstream and exons. To determine the enrichment of transcription factor binding-sites within those chromatin accessible regions, we scanned for transcription factor motifs (TFBS, transcription factor binding sites) using HOMER (findMotifs.pl). Known motif analysis revealed the enrichment of TF binding motifs of Sox2, Nanog and Oct4 (Fig. 4A). We compared ATAC-seq peaks surrounding specific genes of relevance to EMT, cancer cell dormancy and cancer stem cells including Sox2, Oct4, p53, smad2, smad3, c-Myc, STAT3 in the three experimental conditions. The motif discovery tool searches for motifs of binding sites with highest statistical significance for enrichment. The *P* values for enrichment were shown in Supplementary Table 1. As shown in Fig. 4B, we found significant enrichment of TF binding motifs affecting EMT, cellular dormancy and cancer stem cells in dormant and reactivated cancer cells, suggesting a direct binding-activation mechanism (Fig. 4B). We performed peak-calling prioritization pipeline (PePr) to find the differential peaks between cells in 3 states (*P* < 0.05) [29]. The differential peaks between dormant cancer cells and NC cancer cells or between reactivated cancer cells and dormant cancer cells were shown in Supplementary Tables 2 and 3. The differential peaks in these 3 cellular states were highly enriched in cancer-related Ras signaling pathway, Insulin resistance, ABC transporters et al. (Fig. 4C, Supplementary Table 4). We are wondering if the open chromatin regulates the gene expression. We found that the ATAC-seq peaks surrounding the specific genes were associated with their RNA-seq peaks, suggesting that open chromatin patterns play a role in related gene expression (Fig. 4D). We then investigated the relationship between the promoter accessibility of Nanog-targeted genes, Oct4-targeted genes or Sox2-targeted genes and gene expressions of Nanog, Oct4 and Sox2 identified from RNA-seq. The Spearman correlation analysis was performed and the positive correlation was shown between the promoter accessibility of Nanog-targeted genes, Oct4-targeted genes or Sox2-targeted genes and expressions of NANOG, Oct4 and Sox2 (Fig. 4E, Supplementary Table 5). The target genes were selected according to ChIP-binding sites from previously published findings [30]. It demonstrated that the occupancy of these key transcription factor (Oct4, Sox2, Nanog) within chromatin accessible regions determined the transcription of target genes. The integrative genomics viewer (IGV) was used to visualize the differentially accessible regions of Nanog- and Sox2- targeted genes (Fig. 4F). It demonstrated that Nanog and Sox2 are the key transcription factors who regulate the gene expressions during the process of enrichment of cancer stem cells.

### EphB1 and p-EphB1 showed dynamic expression in dormant and reactivated cancer cells

**Fig. 5:**
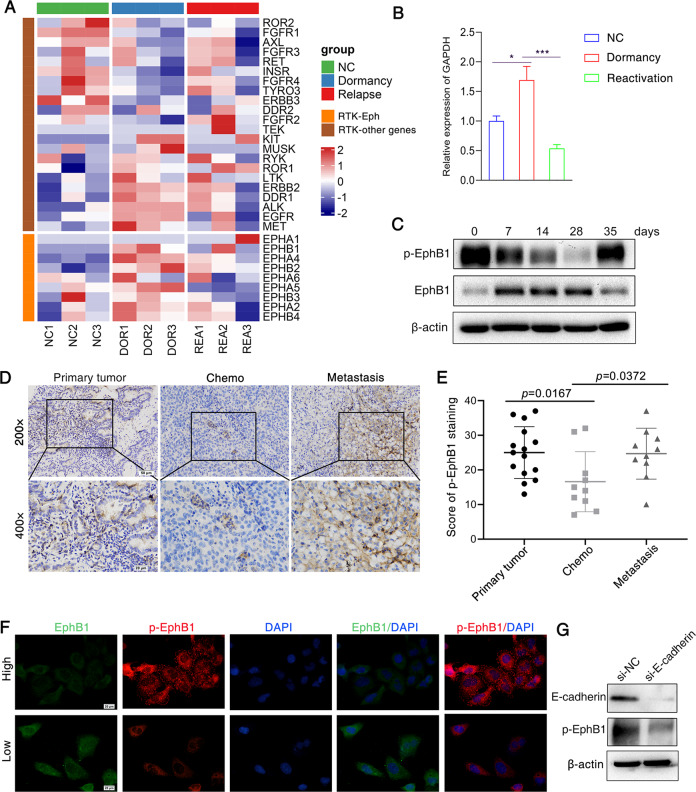
EphB1 and p-EphB1 show dynamic expression in dormant and relapse cancer cells. **A** The heatmap of the expression of receptor tyrosine kinases (RTKs) genes between 3 cell phases induced by cisplatin; The final heatmap was plotted after being balanced by the *Z*-score. *Z* sores were calculated for each row (each gene) by subtracting the mean and dividing by the standard deviation. **B** RT-qPCR Validation of EphB1 expression. **C** Western blot analysis of EphB1 and p-EphB1 in cisplatin-treated A549 cells at different time points; **D**, **E** Assessment of p-EphB1 in lung cancer patients. Tumor biopsies were fixed and stained for p-EphB1. Scale bar = 20 μm; The immunohistochemical scores were shown on the right; Student’s *t* test, *P* values were shown. Primary tumor: biopsies from lung primary tumors; Chemo: biopsies from lung tumors after neoadjuvant chemotherapy; Metastasis: biopsies from metastatic brain of lung cancer patients; **F** Immunofluorescence images of lung cancer cells with different cell density. Cells were planted in 6-well plates at different cell density. Cells at different density were incubated with RFP-conjugated p-EphB1 antibody and GFP-conjugated EphB1 antibody. Cells were counterstained with DAPI. Scale bar = 20 μm; **G** Western blot analysis of proteins obtained from si-E-cadherin-treated A549 cells.

Receptor tyrosine kinases and downstream pathways are druggable targets for cancer treatment. In order to investigate the molecular mechanisms of cisplatin-induced dormancy and reactivation and develop receptor tyrosine kinase inhibitor to reduce therapeutic resistance, we built a heatmap of expressions of RTKs in these 3 status of cells (Fig. 5A). We found that EphBs and EphAs showed dynamic expression during the process of cell dormancy and reactivation (Fig. 5A). We performed Real-time PCR to confirm the dynamic expression of EphB1 in dormant and reactivated cancer cells after chemotherapy (Fig. 5B). We then measured EphB1 expression in lung cancer cells in dormant and reactivated cancer cells by western blot (Fig. 5C). Expression of EphB1 showed an increase in dormant state and a decrease in reactivated lung cancer cell. However, the phosphorylation of EphB1 showed dynamic expression, whose expression was downregulated in dormant lung cancer cells and recovered in reactivated lung cancer cells after chemotherapy (Fig. 5C). It indicated that EphB1 phosphorylation forward signaling was inhibited in the dormant state and promoted in the reactivated state. In order to evaluate the expressions p-EphB1 in lung cancer patients, we collected cancer biopsies from patients who underwent surgery after neoadjuvant chemotherapy and brain metastatic samples of lung cancer patients. The immunohistochemical staining of p-EphB1 revealed that lung cancer biopsies after cisplatin treatment showed decreased p-EphB1. The brain metastatic loci of lung cancer showed increased p-EphB1 (Fig. 5D, E). The discrepancy between EphB1 and phosphorylated EphB1 suggested that the cis signaling or trans signaling of EphB1 dominate in dormant or reactivated cancer cells.

In order to investigate if cell-cell contact triggers the EphB1 signaling, we measured expression of EphB1 and p-EphB1 at different cell seeding densities. Immunofluorescence staining of EphB1 and p-EphB1 showed that p-EphB1 increased in response to cell density (Fig. 5F). Si-E-cadherin downregulated the expression of p-EphB1 (Fig. 5G). It indicated that cell-cell contact induced by E-cadherin is required for the activation of EphB1 forward signal.

### Cis EphB1 signaling promotes migration and dormancy by activating p-p38 and downregulation of E-cadherin

**Fig. 6:**
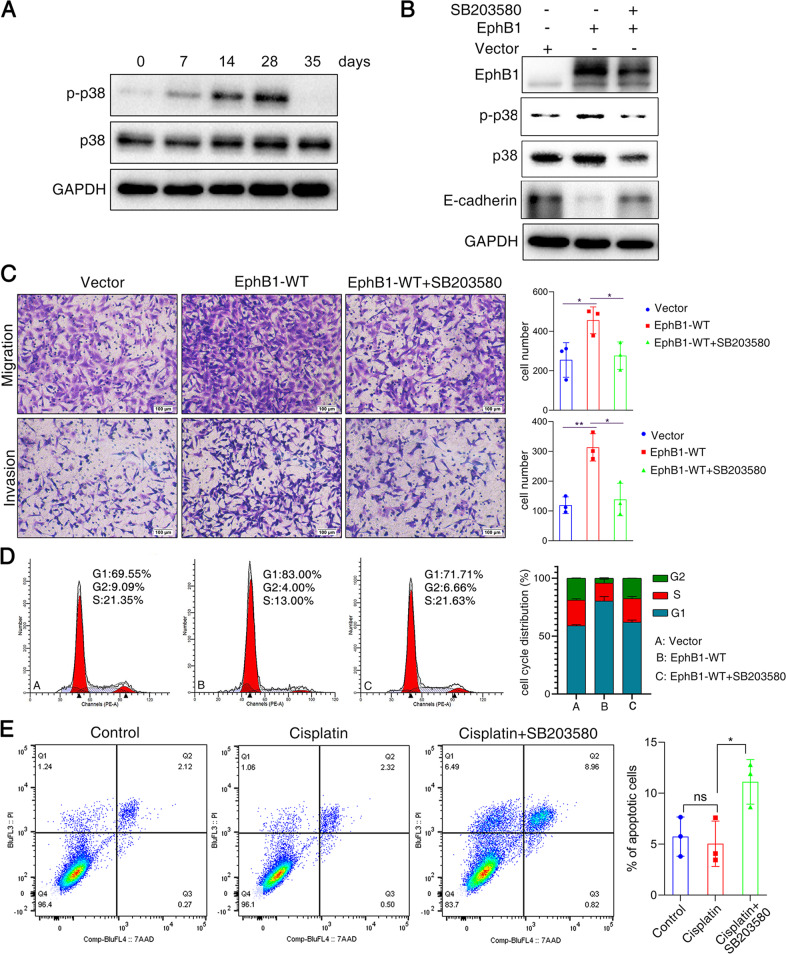
Cis EphB1 signaling mediates cancer cell dormancy after chemotherapy. **A** Western blot analysis of p-p38 in cisplatin-treated A549 cells at different time points; **B** Western blot analysis of proteins obtained from EphB1 transfected cells. A549 cells were transfected with EphB1 with or without treatment of p-p38 inhibitor SB203580. **C** Transwell migration and invasion assays. Representative microscopic images of cells that migrated through or invated through the transwell were shown. Cells with transfected with EphB1 with or without treatment of p-p38 inhibitor SB203580. The histogram graph was shown on the right. Data are presented as the mean ± S.D. from at least three separate experiments. Student’s *t* test, *P value < 0.05, **P value < 0.01; Scale bar = 100 μm; **D** Cell cycle analysis; **E** Flow cytometry analysis of apoptotic cells after treatment of cisplatin combined with p-p38 inhibitor. Representative flow cytometry plots using Annexin V-FITC/PI staining for apoptosis. The percentage of apoptotic cells were shown on the right. Data are presented as the mean ± S.D. from at least three separate experiments. Student’s *t* test, *P value < 0.05, **P value < 0.01.

Considering the upregulation of EphB1 but not phosphorylated EphB1 in dormant state, we focus on the roles of non-phosphorylated cis EphB1 signaling in cellular dormancy. We found that driver of dormancy p-p38 was activated in the cisplatin-induced dormant cancer cells (Fig. 6A). The overexpression of EphB1, which mostly increases the cis signaling, upregulated the expressions of p38 and p-p38 and downregulated the expression of E-Cadherin (Fig. 6B). Inhibitor of p-p38 SB203580 reversed the reduced expression of E-cadherin caused by EphB1, suggesting that EphB1 downregulates E-cadherin through p-p38 (Fig. 6B). Transfection of EphB1 caused the enhanced migration and invasion and SB203580 reversed the increased migration and invasion caused by EphB1(Fig. 6C). Overexpression of EphB1 increased G0/G1 arrest and inhibition of p-p38 reversed the cell cycle arrest, indicating that EphB1 promoted cellular dormancy via p-p38 activation (Fig. 6D). The synergic proapoptotic effect of cisplatin and p-p38 inhibition on dormant cancer cells were assessed by Annexin V assay. The p-p38 inhibitor SB203580 sensitized the dormant cancer cells to cisplatin in vitro and in vivo (Fig. 6E, Supplementary Fig. 3).

**Fig. 7:**
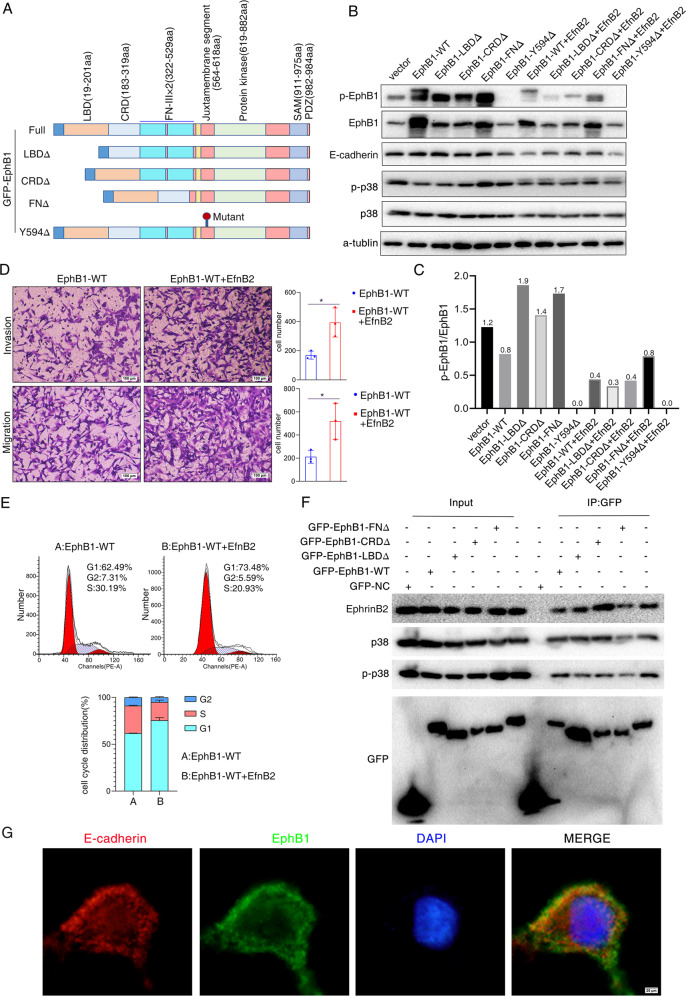
Cis EphB1 signaling regulates cell dormancy and cell contact. **A** Schematic diagram of mutants. Four construct mutants were constructed including mutants that lack the fibronectin type III domain (FNΔ), that lack cysteine-rich region (CRDΔ), that lack ligand-binding domain (LBDΔ) or Y594 phosphorylation site mutation (Y594Δ); **B** Western blot analysis. The expression of EphB1, p-EphB1, p38, p-p38 and E-cadherin was evaluated after transfected with EphB1 wt and mutants or co-transfection of EphB1 and Efnb2; **C** The phosphorylation levels were evaluated by the ratio of phosphorylated EphB1 and total EphB1. **D** Transwell migration and invasion assays after co-transfection of EphB1 and Efnb2; **E** Cell cycle analysis after co-transfection of EphB1 and Efnb2. **F** Protein–protein interactions between EphB1, EfnB2 and p38. GFP/EphB1 was transfected into A549 cells and co-immunoprecipitation was performed using an anti-GFP antibody to characterize protein–protein interactions. **G** Colocalization of EphB1 and E-cadherin. Immunofluorescent images of E-cadherin and EphB1. The transfection of EphB1 constructs expresses fusion GFP. Red fluorescence indicates E-cadherin staining and green fluorescence indicates EphB1. Images were taken at ×100 magnification and scale bar indicates 100 μm.

Considering the forced expression of EphB1 may increase the cis and trans signaling simultaneously, we constructed EphB1 mutants that lack the fibronectin type III domain (FNΔ), that lack cysteine-rich region (CRDΔ), that lack ligand-binding domain (LBDΔ) or Y594 phosphorylation site mutation (Y594Δ) (Fig. 7A). FN, CRD and LBD are reported to be the key domains for Eph cis signaling, being able to form tetrameric complex without the involvement of ephrins. The overexpression of FNΔ, CRDΔ, LBDΔ caused upregulations of p-EphB1 independent of EfnB2 due to the reduction of inhibitory cis signaling (Fig. 7B, line 2,3,4,5). Y594Δ transfection did not show activation of Eph trans signaling (Fig. 7B, line6). Compared to the transfection of WT EphB1, the overexpression of mutants of EphB1 caused downregulation of total EphB1 protein (Fig. 7B, line 2,3,4,5). The phosphorylation levels were evaluated by the ratio of phosphorylated EphB1 to total EphB1 (greyscale values of western blot, Fig. 7C). The increase in phosphorylation levels of EphB1 caused by transfection of mutants confirmed the roles of these domains in cis signaling. We found that the inhibition of cis EphB1 signaling caused by these 3 domains deletion led to upregulation of E-cadherin (Fig. 7B). On the other hand, inhibition of trans EphB1 signaling caused by Y594 deletion decreased the expression of E-cadherin (Fig. 7B). The overexpression of CRDΔ and LBDΔ caused decreased phosphorylation of p38. Co-transfection of EfnB2 with EphB1 caused obvious inhibition of p-EphB1 and the lack of these 3 domains can reverse the inhibitory effects, suggesting that these domains are required for EphB1 cis signaling (Fig. 7B, line 7,8,9,10). On the other hand, transfection of Y594Δ with or without co-transfection of EfnB2 both caused obvious inhibition of EphB1 trans signaling (Fig. 7B, line 6, 11). To directly visualize if co-expressed EphB1 and EfnB2 in the same cell can form complex, we co-transfect GFP/EphB1 and RFP/EfnB2 in 293 cells. We found that EfnB2 and EphB1 in the same cell showed little overlapping distribution and were observed in separate clusters (Supplementary fig. 4). It suggested that EphB1 and EfnB2 in the same cell can promote the Eph-Eph interaction, not Eph-ephrin interaction. We found that co-transfection of EphB1 and EfnB2 caused obviously decreased expression in E-Cadherin (Fig. 7B). The enhanced cellular migration, invasion and cellular arrest in G0-G1 phase were observed in co-transfected A549 cells compared to that of transfection of EphB1 alone (Fig. 7D, E). The enhanced migration, invasion and cellular arrest may be due to downregulation of E-cadherin and increased phosphorylation of p38 by EphB1 cis signaling. From above, it demonstrated that EphB1 cis signaling downregulates the expression of E-cadherin and EphB1 cis signaling countparts trans signaling. We then further investigated if EphB1 binds E-cadherin and p38. We transfected GFP/EphB1 in A549 cells and performed co-immunoprecipitation using an anti-GFP antibody to characterize protein–protein interactions between EphB1, EfnB2 and p38. We found that the binding of EphB1 and ligand Efnb2 was enhanced after CRD and LBD deletion, suggesting the inhibitory effect of these domains on EfnB2-induced trans signaling (Fig. 7F, line 9,10). However, the FN deletion caused reduction in the binding of EfnB2 with EphB1 (Fig. 7F, line 11). It suggested that FN domain may be important for binding of EphB1 with other Ephrin ligand than EfnB2. The binding of EphB1 and p38 was reduced in A549 cells transfected with mutants, suggesting that EphB1 cis signaling mediates the proteolytic cleavage of p38 (Fig. 7F, line 9,10,11). The immunofluorescence analysis was performed to investigate the expression and co-localization of E-cadherin and EphB1 (Fig. 7G). The transfection of CRDΔ and LBDΔ caused enhanced expression of E-cadherin and enhanced location of E-cadherin at cellular plasma and membrane compared to transfection of EphB1 wt (Supplementary Fig. 5). The transfection of FNΔ and Y594Δ did not show obvious upregulated expression of E-cadherin (Supplementary Fig. 5). In this part, we found that EphB1 cis signaling obviously activated p-p38 and followed by downregulation of E-cadherin.

### EphB1 forward signal promotes cancer stem cell enrichment via trans signal

**Fig. 8:**
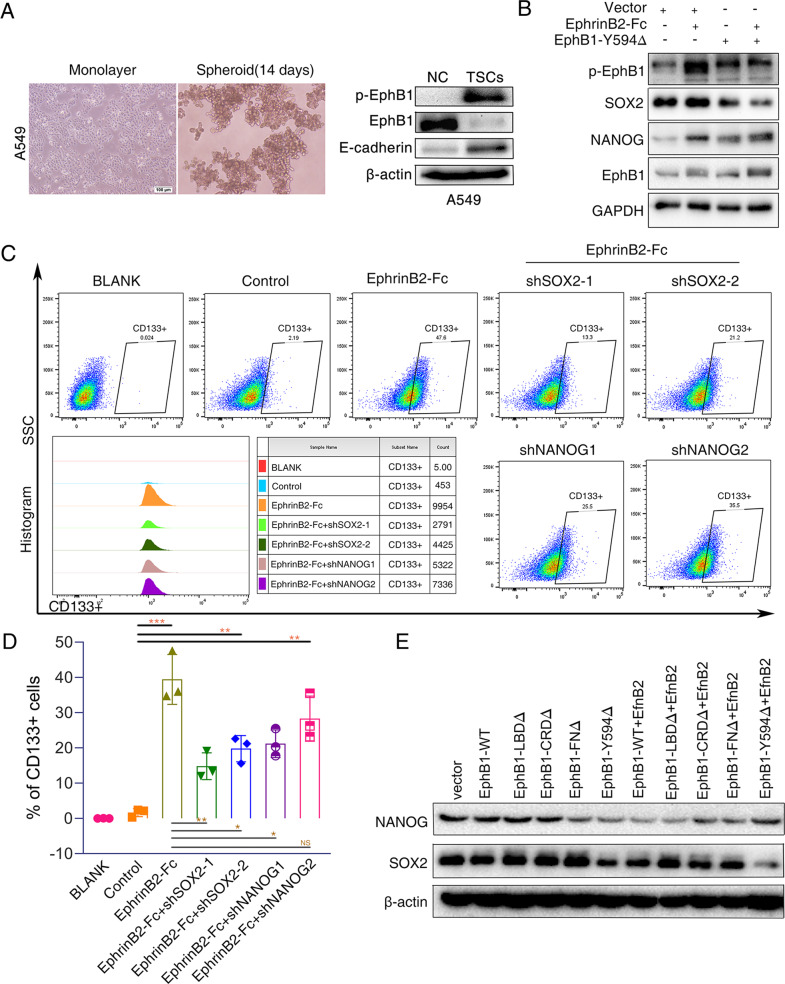
Trans EphB1 signaling promotes cancer stem cell enrichment. A549 cells were seeded at low density in sphere medium in ultra-low attachment cell plates. Formed spheres were observed after 14 days. **A** Representative images of sphere-forming cells. Western blot assay was used to evaluate the expression EphB1, p-EphB1 and E-cadherin in sphere-forming cells. **B** Western blot analysis of cells after treatment of EphrinB2-Fc. **C** Flow cytometry analysis for stem cell surface marker CD133. The histogram graph was shown in (**D**). **E** Western blot analysis. The expression of Nanog and Sox2 was evaluated after transfected with EphB1 wt and mutants or Co-transfection of EphB1 and EfnB2.

We next investigated the roles of phosphorylated EphB1 in the enrichment of cancer stem cells. Cancer stem cells have been isolated from tumor sphere suspension cultures. Western blot assay revealed that p-EphB1 was obviously increased in the sphere cells (Fig. 8A). Of interest, E-cadherin was dramatically increased in the sphere cells (Fig. 8A). We treated cells with EphrinB2-Fc which mediated the trans signaling through phosphorylation of EphB1. We found that phosphorylation of EphB1 dramatically increased the expressions of Sox2 and Nanog (Fig. 8B). The treatment of EphrinB2-Fc on A549 cells transfected of Y594Δ caused reduced expression of Sox2 and Nanog compared to A549 cells transfected with EphB1 WT, confirming the role of EphB1 phosphorylation trans signal on cancer stem cell enrichment (Fig. 8B). The treatment of EphrinB2-Fc on A549 cells increased the ratio of CD133^+^ cancer stem cells and knockdown of Sox2 and Nanog by siRNA could reverse the enrichment of cancer stem cells induced by EphB1 trans signaling (Fig. 8C, D). It indicated that EphrinB2 increased the population of cancer stem cells through Sox2 and Nanog.

To examine the relationship between EphB1 cis- and trans- signalings and enrichment of cancer stem cells, we transfected A549 cells with EphB1 WT and mutants. We found that co-transfection of EphB1 and EfnB2 caused downregulation of Nanog and Sox2. The expression of Sox2 and Nanog was strongly correlated with trans signaling and negatively correlated with cis signaling, which are consistent with the expression profiles of Nanog and Sox2 in dormant and reactivated states (Fig. 8E).

## Discussion

Dormancy, EMT and plasticity of CSCs are interconnected processes [31]. Activation of the EMT program, eliciting the mobility of cancer cells, can acquire stem-like traits [31]. In this study, dormant cancer cells induced by cisplatin shared overlapping characteristics with cancer stem cells and cells underwent EMT. Increased expression of EphB1 and decreased expression of p-EphB1 suggested that reduction of cell-cell contact in dormant cancer cells disrupted EphB1 trans signaling triggered by opposing cells and promoted EphB1 cis signaling which occurred in the same cells. Compared with dormant cancer cells, reactivated cancer cells are characterized by higher expression of cell-cell adhesion molecule E-cadherin. The cell-cell adhesion can trigger the trans signaling of EphB1.

It has been noticed for a long time that a pause- or lag time exists between dissemination and metastatic outgrowth in clinical patients and experimental mouse models [32]. The disruption of E-cadherin-mediated cell-cell contact leads to dysfunction in maintaining epithelial barriers and induces EMT. Primary cancer cells undergo an EMT and enter into the circulation. These disseminated tumor cells (DTCs) are known dormant cancer cells and eventually exit from cellular quiescence to initiate metastatic growth [32]. The revert of epithelial properties following mesenchymal-epithelial transition (MET) enable proliferative growth and induce pluripotent stem cell generation [33, 34]. Although E-cadherin has been considered to be a tumor suppressor gene, continuous expression of E-cadherin in metastasis has been reported [35]. Many metastases express E-cadherin and functional state of E-cadherin in response to environmental factors is an important determinant of metastatic potential instead of the expressional states [36]. E-cadherin-mediated cell-cell contact promotes the formation of circulating tumor cell (CTC) cluster. CTC cluster show selective advantages versus single CTCs [37]. In this study, the surviving lung cancer cells after treatment of cisplatin displayed cancer stem cell properties concomitant with transient EMT-MET switches. The loss and revert of epithelial properties during the process EMT and MET conferred the lung cancer cells selective advantages. Instead of being back to initial status of cancer cells, the cells underwent EMT-MET enriched cancer stem cells. We speculated that cell adhesion-mediated sphere formation was involved in the enrichment of cancer stem cells and cell adhesion-mediated sphere formation triggered EphB1 trans signaling. The precise role of cell-cell contact mediated by E-cadherin needs to be further evaluated.

**Fig. 9:**
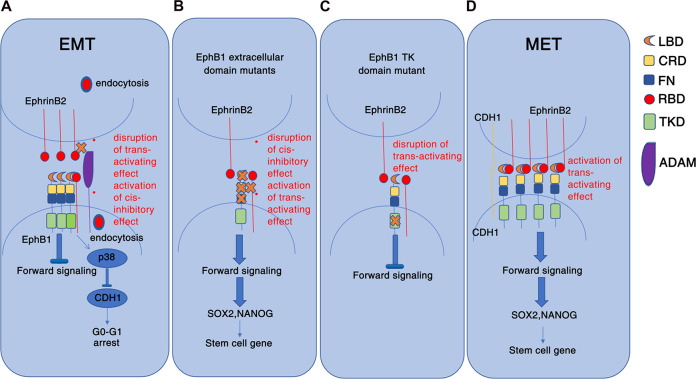
Schematic mechanism of EphB1 cis and trans signaling controlling the cell dormancy and reactivation after chemotherapy. **A** In the dormant EMT cells which showed disrupted cell-cell contacts, ligand-independent EphB1 promoted entry of lung cancer cells into G0-G1 phase of cell cycle through activating p-p38 and downregulating E-cadherin; **B** Interaction of cell-contact-mediated cis- and trans-signalings. Disruption of cis-signaling activates trans-signaling; **C** Disruption of trans-signaling inhibits EphB1 forward signaling; **D** In the state of MET, in which cell-cell adhesion was recovered, interactions of EphB1 and ligand EphrinB2 in trans promoted the stemness of cancer cells through upregulating Nanog and Sox2.

The classic model of Eph and ephrin function involves ephrins acting as in trans ligands of Eph receptors in opposing cells, resulting in “forward” signaling [38]. The paradox roles of EphB1 on the cancer progression remind researchers the diverse function of EphB1. Aberrantly overexpression of EphB1 in varied of cancers leads to the hypothesis that upregulated expression of Eph receptors have functions independent of ephrins [18]. The major difficulties of studying Eph signaling is because ephrins that was expressed in the same cells with Eph receptors may promote inhibitory cis effects on forward phosphorylated signalings. Another question is the extent to which Eph phosphorylated and non-phosphorylated functions play roles in the cancer progression. The Eph-Eph interaction which forms linear arrays of staggered parallel receptors triggers signaling independent of Ephrin ligands [39]. The specific proteolytic cleavage of Eph in cell-cell contacts depends on the ability of the receptor to form cluster [21]. In our study, phosphorylated EphB1 and non-phosphorylated EphB1 conferred different functions in the dormancy and reactivation of lung cancer cells after treatment of cisplatin. In dormant state of cisplatin-treated dormant A549 cells, EphB1 exhibited non-phosphorylated functions and activated p38 MAPK pathway due to lack of trans signaling from opposing cells. The p38 MAPK pathway is well known for its capacity to induce dormancy in tumor cells through downstream effector MSK1. The inhibition of p38 induces tumor proliferation, mediating molecular switching between proliferative and dormant states in tumors [40, 41]. The p38 MAP kinase and a p38-interacting protein (p38IP) are required for downregulation of E-cadherin during the EMT in gastrulation [42]. In this study, the kinase-inactive EphB1 mutant which is lack of 594/600 tyrosine also activated p38 MAPK pathway and also exerted a dominant-negative effect that inactivated EphrinB2 mediated EphB1 phosphorylation and decreased the expression of Nanog. Non-phosphorylated EphB1 activated p38 signaling and degraded E-cadherin. The abolished cis signaling enhanced E-cadherin in the complex and the regain of cell-cell contact in MET reactivated cancer cells resulted in the activation of EphB1 trans signaling triggered by opposing cells. In these phases, EphB1 exhibited phosphorylated functions and upregulated the expression of Sox2 and Nanog (Fig. 9). Co-expressed ephrin ligands can promote Eph receptor oligomerization in cis and inhibit the forward signaling [23]. In our study, we found that co-expressed EphrinB2 which activates EphB1 in cis obviously inhibited expression of E-cadherin. The FN type III domain mutant with repaired cis signaling caused increased trans signaling and followed increased E-cadherin, Nanog and Sox2. Our results indicate that EphB1 ligand dependent and independent signaling play pivotal roles in regulating tumor dormancy and reactivation (Fig. 9).

## Materials and methods

### Antibodies and reagents

Antibodies to proteins were obtained from the following sources: EphB1 (#ab129103) and phos-EphB1 (#ab61791) for Western blot were purchased from Abcam; EphB1 (#AF542) for immunohistochemistry was purchased from Novus Biologicals; Ki67, p53, CyclinA2 and E-cadherin (#3195) were from Cell Signaling Technology; β-actin (#60008-1-Ig) and GAPDH (#60004-1-Ig) were from Proteintech. Fluorescence-labeled ki67 and CD133 were purchased from Abcam, USA. Recombinant human Ephrin-B2 Fc chimera protein was purchased from R&D (7397-EB, RD Inc, MN, USA). Cisplatin (P4394) was purchased from SigmaAldrich (Sigma, MO, USA). Cells were treated with cisplatin at 10 μg/ml for A549 for 48 h.

### Cell culture, plasmid construction, siRNA and patients

NSCLC cell lines A549, H460 and murine Lewis lung cancer cells LLC were cultured in RPMI-1640 medium supplemented with penicillin G (100 U/mL), streptomycin (100 mg/mL) and 10% fetal calf serum. Cells were grown at 37 °C in a humidified atmosphere of 5% CO_2_ and were routinely sub-cultured using 0.25% (w/v) trypsin-EDTA solution.

A full-length EphB1, RBD domain deletion, CRD domain deletion, FN domain deletion were cloned into pEGFP-N1 plasmid in frame with the GFP coding sequence. EphB1 Y594 mutant was generated using site-directed mutagenesis with the Mut Express II Fast Mutagenesis Kit V2 (Vazyme, #C214). The primers used to construct plasmids are as follows: 5′-GATGAAGATCTGCA TTGACCCCTTCACTTACGAGGATCCC-3′; 5′-AGGGGTCAATGCAGATCTTCATCCCTGGGGAGCCTCGGCC-3′. si-E-cadherin: 5′-CTCCGTTTCTGGAATCCAA-3′ and negative control (NC) siRNA duplexes were purchased from Ribobio (Guangzhou, China). Plasmids and siRNAs were transfected into cells using Lipofectamine 3000 (Invitrogen).

Patients diagnosed with NSCLC (*n* = 35) were included in this study, 15 of which were diagnosed with lung cancer without treatment and 10 of which were diagnosed with lung cancer after cisplatin based neoadjuvant chemotherapy and 10 of which were diagnosed with lung cancer with brain metastasis. All cases enrolled in this study were diagnosed at the second Xiangya hospital, Central South University, China. The clinicopathological characteristics of the cases are summarized in Table 1. The patients were informed of the sample collection and signed informed consent forms. The collection and use of samples were approved by the ethical review committees of the second Xiangya Hospital, Central South University.

**Table 1:** Clinical characteristics of 35 patients.

Clinical characteristics of patients	
Patients (*n* = 35)	
NSCLC patients without neoadjuvant chemotherapy	15 (43%)
Underwent surgery after neoadjuvant chemotherapy	10 (28.5%)
Brain metastatic patients	10 (28.5%)
Genders (*n* = 35)	
Male	16 (45.7%)
Female	19 (54.3%)
Age (*n* = 35)	
≤50	10 (28.6%)
>50	25 (71.4%)

### Western blotting

The protein lysate used for western blotting was extracted using RIPA buffer (Biotime, Hangzhou, China) containing protease inhibitors (Roche, Basel, Switzerland). Proteins were quantified using the BCA^TM^ Protein Assay Kit (Pierce, USA). A western blot system was set up using a Bio-Rad Bis-Tris Gel system, according to the manufacturer’s instructions (Bio-Rad, CA, USA). The cell protein lysates were separated on 10% SDS-polyacrylamidegels and electrophoretically transferred to polyvinylidene difluoride membranes (Millipore, Danvers, MA, USA). The primary antibody solution was prepared in 5% blocking buffer. Primary antibodies against EphB1 (Abcam, USA), p-EphB1(Abcam, USA), E-cadherin, p53, Sox2, Nanog et al were incubated with the membrane at 4 °C overnight, followed by a brief wash and incubation with secondary antibody for 1 h at room temperature. An anti-GAPDH or anti-β-actin antibodies were purchased from Proteintech (Chicago, USA) and used as loading controls. Finally, a 40:1 solution of peroxide and luminol was added to cover the blot surface for 5 min at room temperature. The chemiluminescent signals were captured, and the intensity of the bands was quantified using a Bio-Rad ChemiDoc XRS system (Bio-Rad, CA, USA).

### Cell migration and invasion assays

Cell migration and invasion assays were both performed using a transwell insert that contains polycarbonate filters with 8-μm pores (cat. no. 3422; Corning). Cells (5 × 10^4^) were suspended in 200 µl of serum-free medium and added to the transwell membrane in the upper chamber. Migrated cells were fixed in 4% paraformaldehyde and stained with crystal violet. Migrated cell images were observed and imaged under microscope (CKX41, Olympus, Japan). Cell migration was quantitated by counting in 10 random fields on the lower membrane surface. Invasion capacity of cells was measured by Matrigel matrix gel invasion assay. The surface of the filter (8-μm pore size) of the upper chamber was coated with 1 mg/ml Matrigel matrix. Cells (5 × 10^4^) were suspended in 200 µl of serum-free medium and added to the transwell membrane in the upper chamber. Invaded cells were fixed in 4% paraformaldehyde and stained with crystal violet. Cell invasion was observed and imaged under microscope (CKX41, Olympus, Japan). Cell invasion was quantitated by counting in 10 random fields on the lower membrane surface.

### Immunohistochemistry

Lung biopsies and brain metastatic biopsies were fixed and embedded in paraffin wax. Four- to six-μm thick paraffin sections were defaced followed by hydration. Tissue sections were incubated with p-EphB1 antibody at 4 °C overnight in a humidified chamber. After extensive washing with PBS, sections were incubated with biotin-linked goat anti-rabbit IgG antibodies (UltraSensitive S-P Kit, Maixin Biotechnology Company, Fuzhou, China). The sections were then washed and followed by developing in 3′-diaminobenzidine hydrochloride (DAB) as chromogen, and sections were counterstained with haematoxylin. Finally, after dehydration and mounting, the sections were observed and imaged under microscope (OLYMPUS BX-51, Japan). Goat serum and PBS were used instead of the first antibody as a negative control and blank control respectively. A semi-quantitative scoring criterion for IHC was used in which both the staining intensity and positive areas were recorded.

### Quantitative real-time PCR

Total RNA was extracted from the cells using Trizol (Invitrogen, Carlsbad, CA, USA) according to manual instruction. cDNA was synthesized from total RNA using the RevertAid First Strand cDNA Synthesis Kit (Thermo Scientific, Waltham, MA, USA). GAPDH was used as the endogenous control. Quantitation PCR was performed according to the indications. Real-time PCR was performed using the Bio-Rad IQTM5 Multicolor Real-Time PCR detection System (Bio-Rad, Berkeley, CA, USA). Relative mRNA expression levels were calculated by the 2^−ΔΔCt^ method. The primers for real-time PCR are shown in Table 2 [17].

**Table 2:** Primer sequence for Real-Time PCR.

Gene	Forward primer (5′- to 3′)	Reverse primer (5′- to 3′)
*EphB1*	ATGCGCTTCACTGTGAGAGAC	ATTCCGAGTAAGAGGCCCAAA
*Snail*	TCAAGATGCACATCCGAAGCC	TTGTGGAGCAGGGACATTCG
*Slug*	AGATCTGCCAGACGCGAACT	GCATGCGCCAGGAATGTTCA
*CDH1*	TGAAGCCCCCATCTTTGTGC	GGCTGTGTACGTGCTGTTCT
*GAPDH*	AACGGATTTGGTCGTATTGG	TTGATTTTGGAGGGATCTCG

### RNA sequencing platform technologies and pipelines

Total RNA was extracted from the cells using Trizol (Invitrogen, Carlsbad, CA, USA) according to manual instruction. Total RNA was qualified and quantified using a Nano Drop and Agilent 2100 bioanalyzer (Thermo Fisher Scientific, MA, USA). Total RNA was isolated from cancer cells and cDNA library was constructed. Briefly, Oligo(dT)-attached magnetic beads were used to purify long transcripts such as mRNA. Purified mRNA was fragmented into small pieces with fragment buffer at appropriate temperature. Random hexamer-primed reverse transcription was performed to synthesize double-stranded cDNA. The cDNA fragments were amplified by PCR, and products were purified by Ampure XP Beads, then dissolved in EB solution. The product was validated on the Agilent Technologies 2100 bioanalyzer for quality control. The final library was amplified with phi29 to make DNA nanoball (DNB) which had more than 300 copies of one molecular, DNBs were loaded into the patterned nanoarray and single end 50 bases reads were generated on BGIseq500 platform (BGI-Shenzhen, China). The sequencing data was filtered with SOAPnuke (v1.5.2) [43] by (1) Removing reads containing sequencing adapter; (2) Removing reads whose low-quality base ratio (base quality less than or equal to 5) is more than 20%; (3) Removing reads whose unknown base (‘N’ base) ratio is more than 5%, afterwards clean reads were obtained and stored in FASTQ format. The clean reads were mapped to the reference genome (Homo_sapiens, GCF_000001405.38_GRCh38.p12) using HISAT2 v2.0.4) [44, 45]. Bowtie2 (v2.2.5) [46] was applied to align the clean reads to the reference transcriptome, then expression level of gene was calculated by Expectation Maximization (RSEM) (v1.2.12) [47]. The expression levels for each of the genes were normalized as fragments per kilobase of exon model per million mapped reads (FPKM) by RSEM. Essentially, differential expression analysis was performed using the DESeq2(v1.4.5) [48] with *Q* value ≤ 0.05. The heatmap of differentially expressed genes with unsupervised clustering was built by pheatmap package (v1.0.8) in R software. The heatmap represented expression values (log2 (FPKM + 1) of differentially expressed genes. After the unsupervised clustering, *Z*-sores were calculated for each row (each gene) by subtracting the mean and dividing by the standard deviation. The final heatmap was plotted after being balanced by the *Z*-score. KEGG enrichment analysis of annotated differential expressed genes was performed by R package ggplot2. The significant levels of pathways were corrected by *Q* value with a rigorous threshold (*Q* value ≤ 0.05). The raw RNA-seq data were deposited in SRA database, with accession number is SRA PRJNA730205.

### Co-Immunoprecipitation (Co-IP)

Co-IP experiments were carried out to examine the protein-protein interaction between EphB1, EfnB2 and p38. Briefly, EphB1 wt/EGFP or EphB1 mutants/EGFP transfected A549 cells were lysed with IP buffer (20 mM Tris, pH 7.5, 150 mM NaCl, 1% Nonidet P-40, 0.1% sodium deoxycholate, 100 mm NaF, 5 mM MgCl2, 0.5 mM Na3VO4, 0.02% NaN3, 0.1 mM 4-(2-aminoethyl)-benzenesulfonyl fluoride, 10 µg/mL aprotinin, and 1 µM pepstatin A). Cell lysates were centrifuged at 22,000 × *g* for 30 min to remove debris. The supernatants were incubated with the agarose beads and then were incubated with 1 µg of specific antibody at 4 °C overnight with gentle mixing. The agarose beads were collected by centrifugation. The non-precipitated supernatant was used as Input. GFP null vectors were used as negative controls. The agarose beads were washed and then mixed with 2× sample loading buffer. The sample was subjected to the Western blot assay to detect EfnB2, p38 and p-p38.

### Fluorescence microscopy

Cells were cultured on glass coverslips for 24 h before treatment. After transfection with GFP-EphB1 WT, the cells were rinsed in tris-buffered saline (TBS; 6% tris, 8.8% NaCl, 85.2% dH2O, pH 7.6) and were fixed by cold methanol for 4 min. Cells were permeabilized with TBS containing 0.2% Triton X-100 for 10 min followed by blocking with TBS containing 1% BSA and 0.1% Triton X-100 for an hour. After blocking, the coverslips were incubated in E-cadherin primary antibody for an hour. The coverslips were rinsed for three times and then incubated in RFP-conjugated secondary antibody for an hour in the dark. The coverslips were then washed and incubated with DAPI. The coverslips were then mounted on glass slides and observed under microscope (OLYMPUS BX-51, Japan).

For the immunofluorescent staining for EphB1 and p-EphB1, cells at different density were incubated with RFP-conjugated p-EphB1 antibody and GFP-conjugated EphB1 antibody. Cells were counterstained with DAPI.

### ATAC-seq

ATAC-seq analysis was performed by BGI-Shenzhen (https://en.genomics.cn). Cells obtained from 3 replicates of 3 stages of cancer cells were harvested and resuspended in cold lysis buffer, followed by spinning down by centrifuge. A crude nuclei was prepared and immediately continued to transposition reaction. Immediately following the nuclei preparation, resuspend the pellet in the transposase reaction mix. The transposase reaction mix was incubated at 37 °C for 30 mins. After transposition, purified DNA was amplified. qPCR based methods were used to quantify ATAC-seq libraries. The libraries were sequenced using Illumina high-throughput sequencing instruments. Genome depth distribution obtained by BEDTools and only those unique mapped reads were used in this analysis. Only the alignments within 2 mismatches were considered in Peak calling. We used MACS2 (version: 2.1.0) to call peaks (open chromatin regions) [49]. IDR (v2.0.4) was applied to measure the reproducibility of findings identified from replicate sample. The differential peak analysis of ATAC-seq was performed by PePr [29]. Briefly, a proper window width was estimated based on the average width of the top peaks. PePr divided the genome into consecutive windows. The reads were counted in each window and the read counts for each window were linearly scaled using a normalization constant estimate for each sample. The differential enrichment peaks from different sample were visualized by IGV. The pathway enrichment of differential peaks was performed based on Kyoto Encyclopedia of Genes and Genomes database. The raw ATAC-seq data were deposited in SRA database, with accession number SRA PRJNA731334.

### Motif analysis

The occupancy of transcription factors within those chromatin accessible regions from ATAC-seq was determined by scanning for transcription factor motifs (TFBS, transcription factor binding sites) using HOMER software. The ATAC sequence Fasta files were used to find motifs and findMotifs.pl was used to seek regulatory patterns of genes. In order to search for Known Motifs, HOMER software loads a list of previously determined motifs. HOMER software scans sequences for motifs and calculates the final enrichment by considering target versus background sequences. The motif with the most significant *p* value predicted by HOMER software was selected as key transcription factors. For each cell type, we used 1 × 10^-8^ as *P* value cutoff. An HTML page was created as the motif output that contains the images.

### Spearman correlation analysis

The findMotifs.pl exerts smaller number for promoter analysis and can be used to discover motifs in promoter regions. We calculated the RPKM of peaks in the 2 kb upstream of transcription start site of Nanog-targeted genes, Oct4-targeted genes or Sox2-targeted genes. Featurecounts (Rsubread package) was used to calculate the counts of reads in peak and personal script was used to normalize the counts of reads in peak to get the RPKM values. The target genes were selected according to ChIP-binding sites from previously published findings [30]. The Spearman correlation analysis was performed between the RPKM of peaks in ATAC-seq and the expressions of Nanog, Oct4 and Sox2 in RNA-seq in those 3 stages of cells.

### Apoptosis assay

Apoptosis assay were performed using an Annexin V-fluorescein isothiocyanate (FITC) apoptosis antibody (BD Bioscience) according to the manufacturer’s instructions. Briefly, cells were plated at a density of 5 × 10^5^ cells/well into 6-well plates overnight at 37 °C. Cells were treated with cisplatin with or without p-p38 inhibitor. After treatment, cells were then pelleted and washed with PBS. The cells were resuspended in 1× binding buffer, followed by the addition of Annexin V-FITC and propidium iodide (PI). Samples were tested using the FACS Calibur flow cytometer. The percentage of apoptotic cells was determined.

### Cell cycle analysis

After treatment, cells were detached and fixed in 70% cold ethanol for 2 h. Cells were then treated with RNAse (1 mg/mL) and Propidium iodide (0.05 mg/mL) was added in the dark for 30 min at 37 °C (Cell Cycle and Apoptosis Analysis Kit, Beyotime, #C1052). The cellular DNA content was evaluated in a FACS flow cytometer (Becton-Dickinson).

### Animal experiment

Animal experiments were conducted following protocols approved by Central South University, China. Six-week-old male C57BL/6 mice were randomly divided into 3 groups. Murine Lewis Lung Cancer cells (LLCs) were subcutaneously injected into syngeneic C57BL/6 mice. The allograft tumor growth was examined using calipers. The volumes were calculated using the following standard formula: length × width^2^ × 0.5. The p-p38 inhibitor SB203580 was intraperitoneally injected at 2 mg/kg.

### Statistical analysis

Data are presented as the mean ± S.D. from at least three separate experiments. Statistical analyses were performed using GraphPad Prism 5 (GraphPad Software, Inc., CA, USA). Multiple group comparisons were performed using ANOVA with a post hoc test for the subsequent individual group comparisons. A *P* value of <0.05 was considered to be significant.

## Supplementary information

aj-checklist

Supplementary figure and table legends

Supplementary Table 1

Supplementary Table 2

Supplementary Table 3

Supplementary Table 4

Supplementary Table 5

Supplementary Fig1

Supplementary Fig2

Supplementary Fig3

Supplementary Fig4

Supplementary Fig5

Supplementary Fig6

## Data Availability

All the data generated or analyzed during this study are included in this published paper and its supplementary files. The RNA-seq data and ATAC-seq data have been submitted to the Gene Expression Omnibus and the data could be accessed by the accession numbers SRA PRJNA730205 and SRA PRJNA731334.
