# Supplementary material for: Cell-cell contact-driven EphB1 cis- and trans- signalings regulate cancer stem cells enrichment after chemotherapy
Source: Cell Death Dis. 2022 Nov 19;13(11):980. doi: 10.1038/s41419-022-05385-5 (PMC9675789; doi:10.1038/s41419-022-05385-5)
Supplement: Supplementary file 2 — Supplementary figure and table legends [file 41419_2022_5385_MOESM2_ESM.docx]

**Supplementary figure and table legends**

**Supplementary figure legends**

**Supplementary Figure 1. Cisplatin induces cellular dormancy and reactivation and EMT-MET transition.** Murine lung cancer cells LLC and human NSCLC lung cancer cells H460 were subjected to short-term exposure to cisplatin. Lung cancer cells were treated with cisplatin for 48 hrs. Cisplatin was then withdrawn. The treatment protocol and the microscope images treated with cisplatin at different time points in LLC(A) and in H460(B); (C) Cell cycle analysis of dormant and reactivation cancer cells induced by cisplatin; (D) Assessment of Ki67 in lung cancer patients. Tumor biopsies were fixed and stained for Ki67. Scale bar=50μm. Primary tumor: biopsies from lung primary tumors; Chemo: biopsies from lung tumors after neoadjuvant chemotherapy; Metastasis: biopsies from metastatic brain of lung cancer patients

**Supplementary Figure 2. Differentially expressed genes in 3 stages of cancer cells after treatment of cisplatin.** (A)The genome mapping ratio of 3 stages of cancer cells. Volcano Plot for DEGs between dormant cancer cells and untreated cancer cells (B); between reactivated cancer cells and dormant cancer cells (C); (D)Pearson’s correlation coefficient of gene expression among 3 stages of cancer cells. *P*<0.05. (E) A heatmap of the DEGs with most fold change value. (F) GSEA analysis of differentially expressed genes with FDR<0.25. Gene set enrichment analysis using “cell proliferation” “cell cycle” “cell adhesion” “p53 pathway” was performed. *P*-values, FDR and NES were shown.

**Supplementary Figure 3. Tumor size in syngeneic tumor models.**

LLCs were subcutaneously injected into syngeneic C57BL/6. Mice were given cisplatin (30mg/Kg/day i.p.) for 7 days with or without SB203580. Tumor size was measured twice a week using calipers. The tumor weight was shown on the right.

**Supplementary Figure 4. Distribution of EphB1 and EfnB2 after co-transfection.**

**Supplementary Figure 5. Colocalization of EphB1 and E-cadherin.**

Immunofluorescent images of E-cadherin and EphB1. The transfection of EphB1 constructs expressed fusioned GFP. Red fluorescence indicates E-cadherin staining and green fluorescence indicates EphB1. Images were taken at 100X magnification and scale bar indicates 100 μm.

**Supplementary Figure 6. Original images of representative western blots in all the Figures.**

**Supplementary table legends**

**Supplementary Table 1. Knowmotif Results**

**Supplementary Table 2. DiffGrouppeak-NC_vs_Dormancy_diff_peaks**

**Supplementary Table 3. DiffGrouppeak- Dormancy_vs_Reactivation_diff_peaks**

**Supplementary Table 4. DiffGroupPeakanno-pathway**

**Supplementary Table 5. Spearman correlation analysis**
