# Supplementary figures and images for: Cell-cell contact-driven EphB1 cis- and trans- signalings regulate cancer stem cells enrichment after chemotherapy
Source: Cell Death Dis. 2022 Nov 19;13(11):980. doi: 10.1038/s41419-022-05385-5 (PMC9675789; doi:10.1038/s41419-022-05385-5)

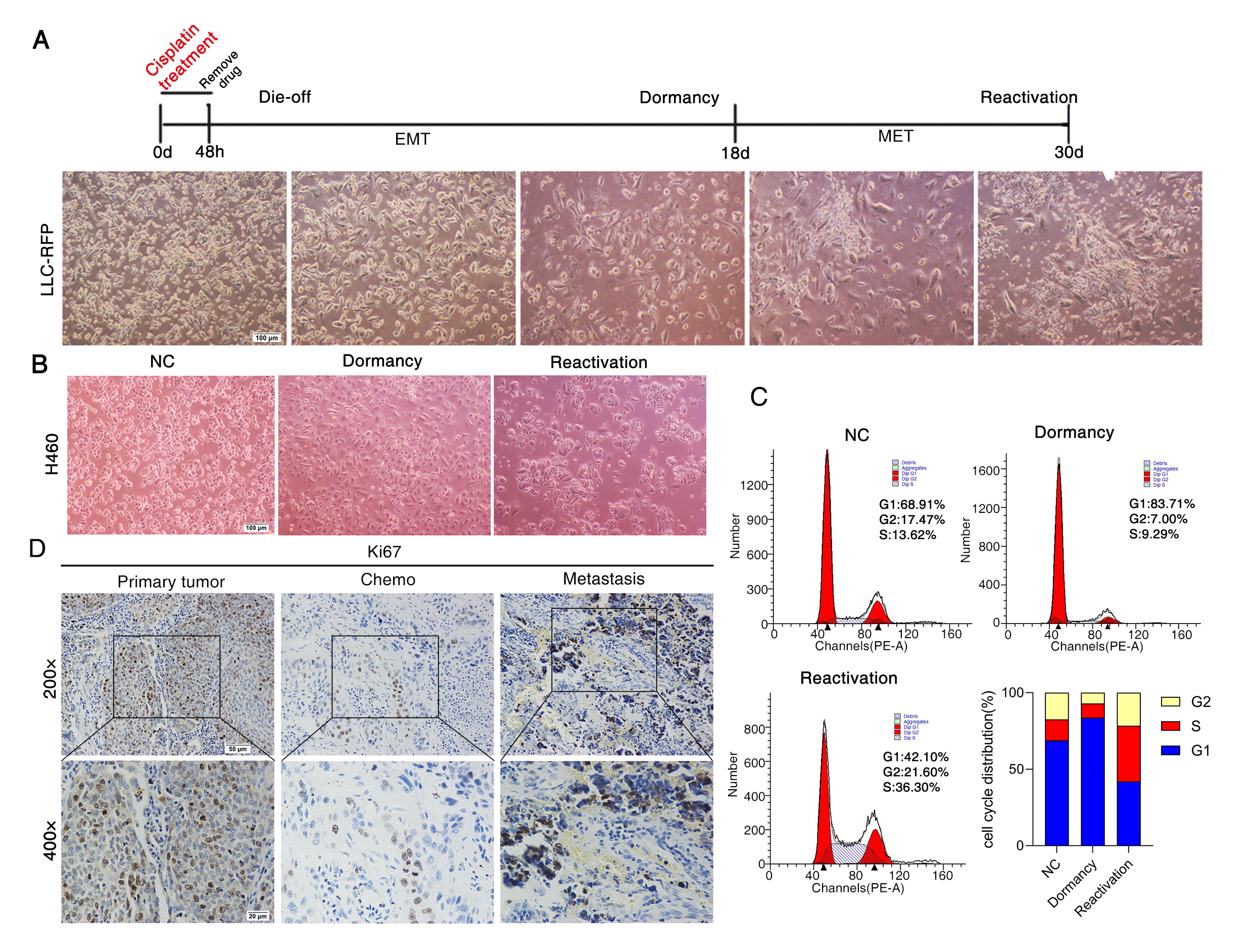

Supplement: Supplementary file 8 — Supplementary Fig1 [file 41419_2022_5385_MOESM8_ESM.tif]

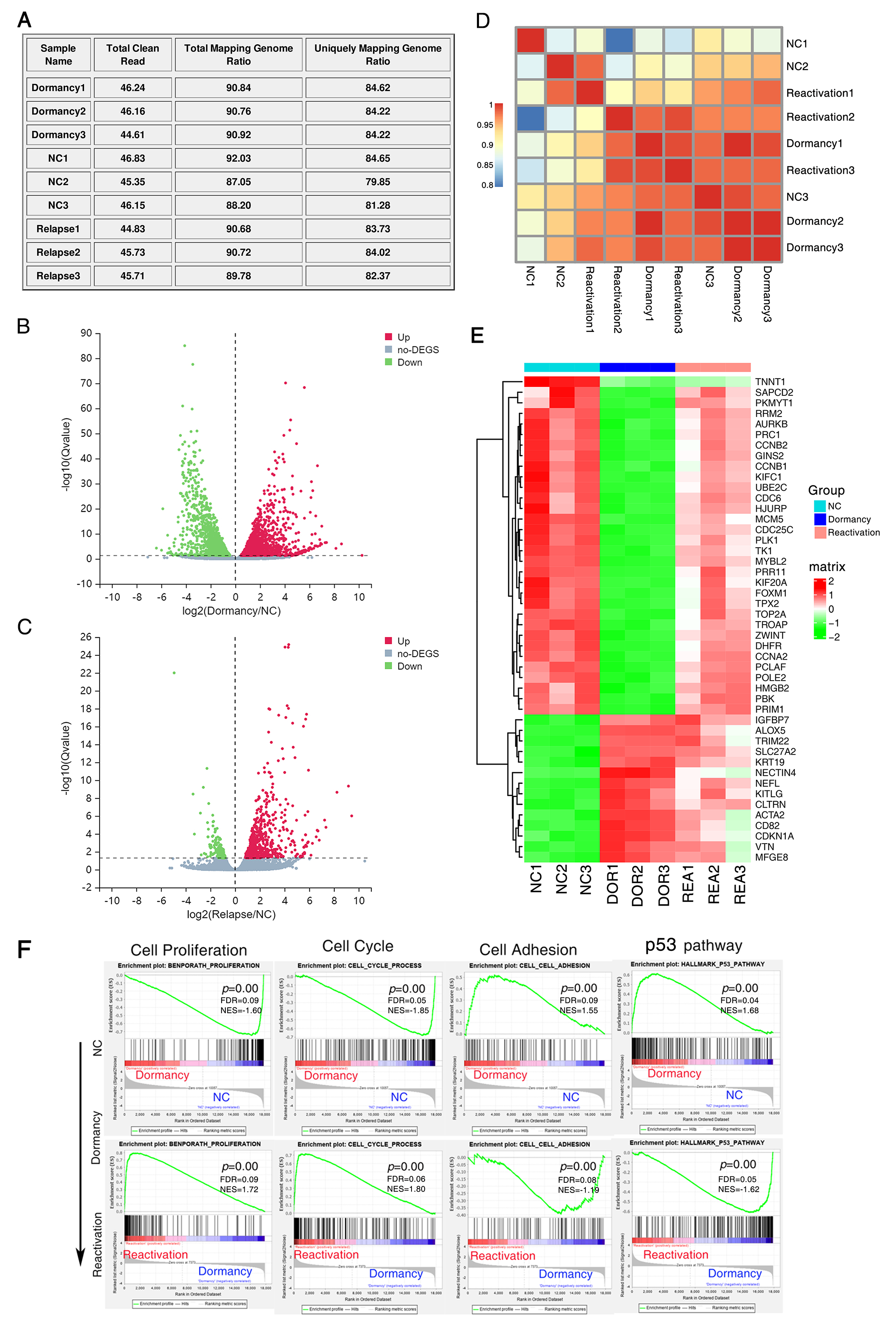

Supplement: Supplementary file 9 — Supplementary Fig2 [file 41419_2022_5385_MOESM9_ESM.tif]

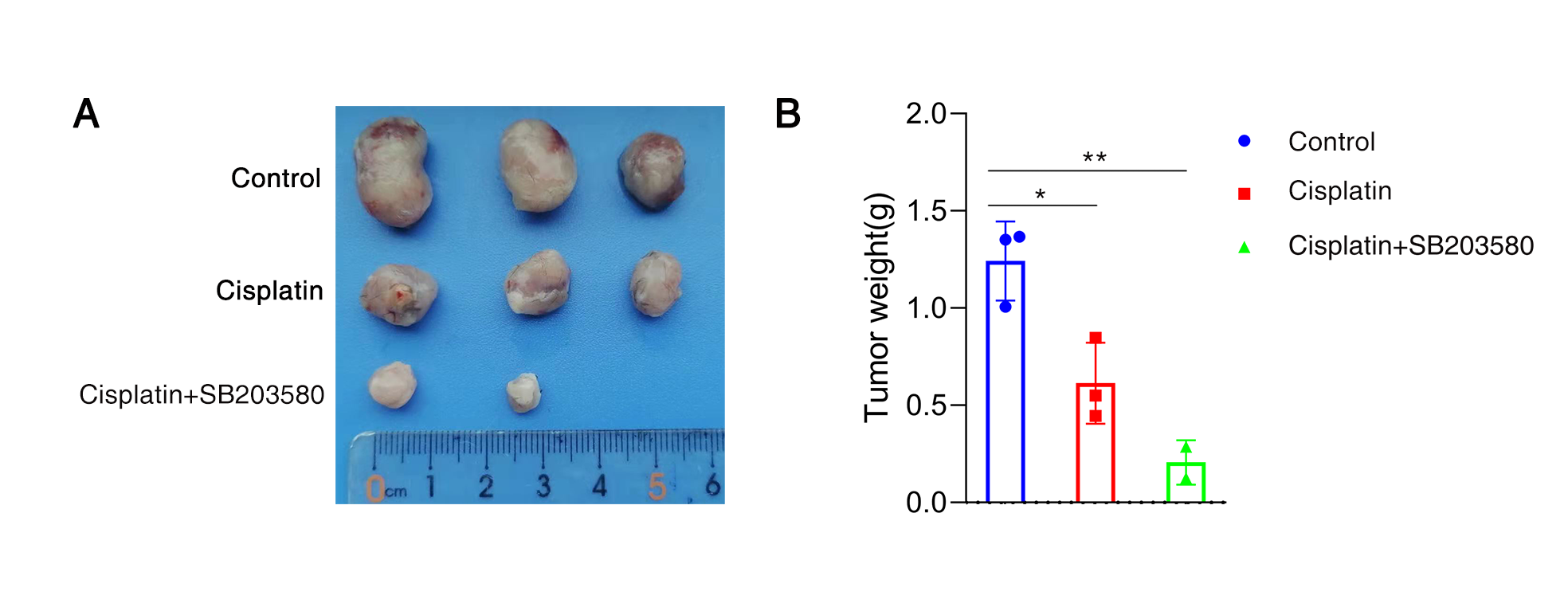

Supplement: Supplementary file 10 — Supplementary Fig3 [file 41419_2022_5385_MOESM10_ESM.tif]

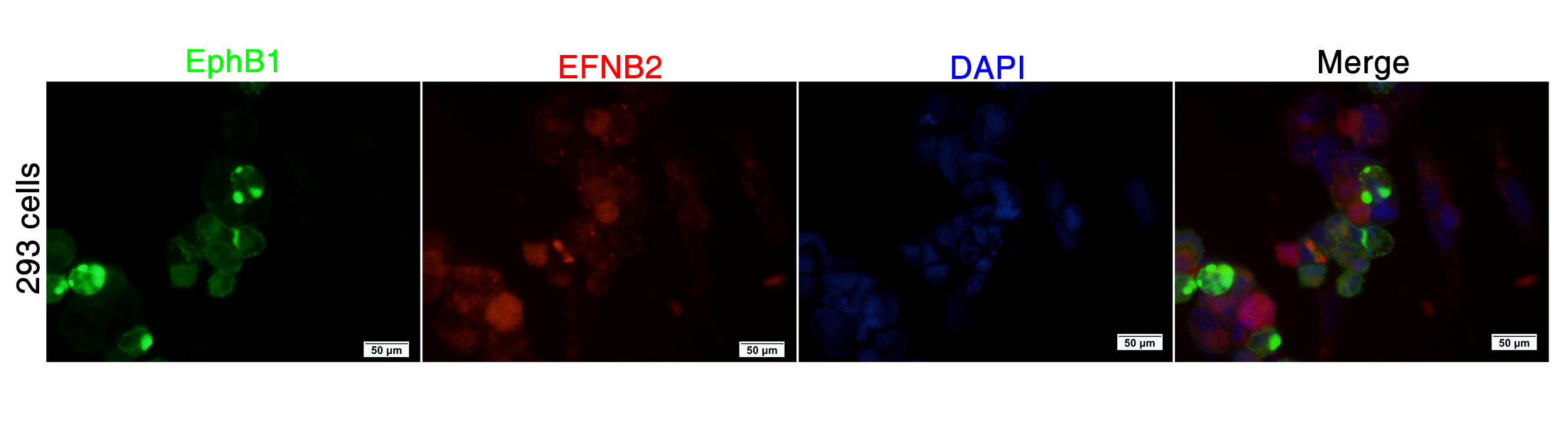

Supplement: Supplementary file 11 — Supplementary Fig4 [file 41419_2022_5385_MOESM11_ESM.tif]

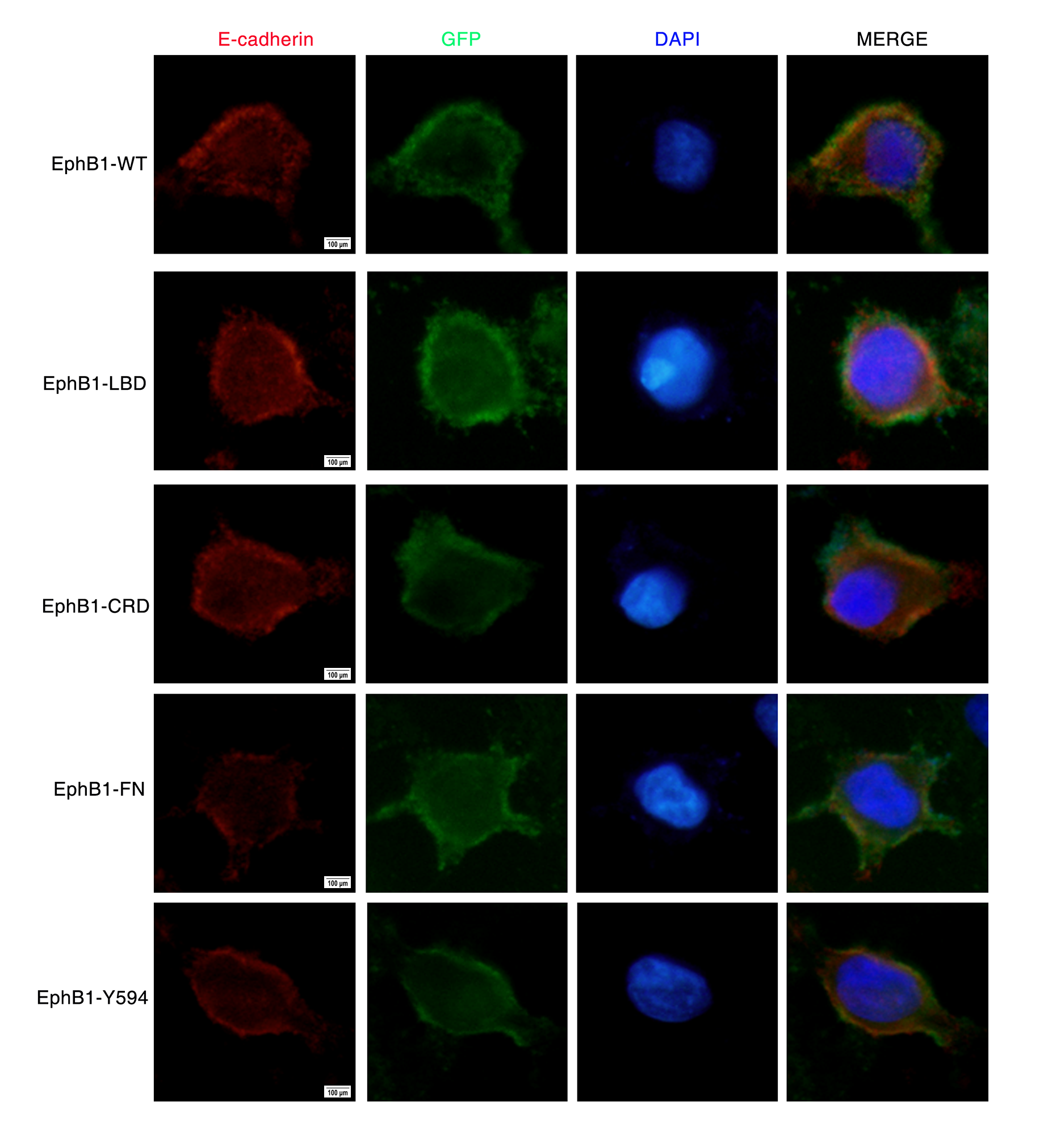

Supplement: Supplementary file 12 — Supplementary Fig5 [file 41419_2022_5385_MOESM12_ESM.tif]

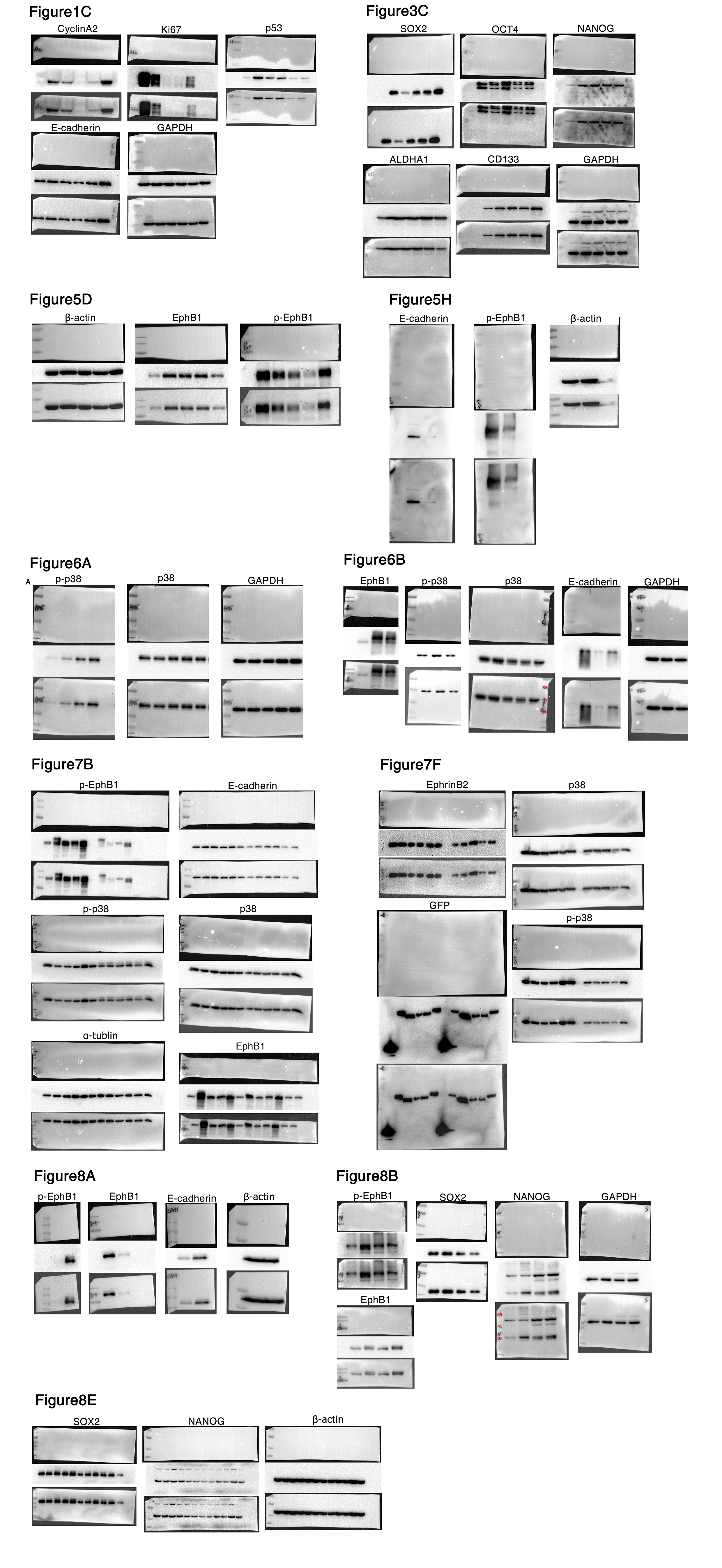

Supplement: Supplementary file 13 — Supplementary Fig6 [file 41419_2022_5385_MOESM13_ESM.tif]
